# Associations between migration experience and perceived mental health in optimal ageing: Evidence from the Sardinian Blue Zone

**DOI:** 10.1002/ijop.12810

**Published:** 2021-09-22

**Authors:** Maria Chiara Fastame, Ilaria Mulas, Marilena Ruiu

**Affiliations:** ^1^ Department of Pedagogy, Psychology, Philosophy University of Cagliari Cagliari Italy

**Keywords:** Resilience, Psychological well‐being, Ageing, Blue Zone, Migration

## Abstract

The effect of migration on perceived mental health has not been examined in older migrants after their return to their places of origin. This study was mainly aimed at evaluating the perceived mental health of older people who experienced migration and permanent resident peers living in the Sardinian Blue Zone (i.e. one of the four areas of exceptional longevity in the world). Forty‐eight community‐based older participants (32 males and 16 females) with and without a migration experience were recruited in two villages of the Sardinian Blue Zone and completed a battery of self‐report inventories assessing psychological well‐being, negative mood, and ego resilience. Older individuals who experienced migration reported higher ego resilience and exhibited greater resources used to manage positive emotionality (i.e. openness to life experiences). Moreover, compared to the normative data, both the groups reported higher psychological well‐being and fewer depressive symptoms. Finally, no significant associations were found between the length of migration and each mental health index. In conclusion, resilience seems to represent a psychological trait that helps to manage stressful events and contributes to the preservation of perceived mental health in late adulthood.

A growing body of evidence has highlighted the significant role played by perceived psychological well‐being (e.g. higher life satisfaction, and lower depression) and resilience (i.e. “the capacity to maintain or regain well‐being in the face of adversity”; Ryff, 2014, p. 10) for the promotion of successful ageing (e.g. Bowling & Iliffe, 2011; Golja et al., 2020). Even though, at present a univocal definition of successful ageing is lacking (Cosco et al., 2014; Depp & Jeste, 2006), a research trend has highlighted the contribution of psychological resources for the optimisation of the ageing process. For instance, two studies conducted in Slovenia (Golja et al., 2020) and in San Diego (USA) (Jeste et al., 2013) showed that fewer self‐reported signs of depression were associated with more successful ageing. Additionally, Jeste et al. (2013) also documented the protective effect of resilience in impacting positively successful ageing. Extending this evidence and assuming a cross‐cultural perspective, more recently Blanco‐Molina et al. (2019) conducted a study with a sample of Spanish and Costa Rican older participants. The authors concluded that social participation, perceived physical health, and spirituality (i.e. three crucial dimensions of successful ageing) predict both hedonic and eudaimonic (e.g. personal growth) psychological well‐being.

Furthermore, it has been assumed that studies conducted with long‐lived individuals can significantly contribute to the definition of the link between psychological resources and successful ageing. Concerning this issue, during the last two decades, a further line of research has been developed in the Sardinian Blue Zone, that is, an experimentally validated geographical spot located in the central‐eastern mountainous part of Sardinia which is known for the longevity of its inhabitants and a very high prevalence of a similar number of male and female centenarians (Poulain et al., 2004, 2013). Embracing a psychological viewpoint, one research trend suggests that community‐dwelling older individuals living in the Sardinian Blue Zone reported better psychological well‐being and fewer depressive symptoms than peers living in a rural area of northern Italy (e.g. Fastame, Penna, & Rossetti, 2014; Fastame, Penna, Rossetti, & Agus, 2014) and in the Sardinian urban areas (Fastame et al., 2015; Fastame et al., 2021). Moreover, it has been well‐established that older individuals ageing well in the Sardinian Blue Zone showed depressive symptoms significantly below the Italian national cut‐off, and moreover they displayed greater perceived psychological well‐being than the normative values provided for Italian sexagenarians and older individuals (for a review, see Hitchcott et al., 2018). This pattern of research is consistent with further studies conducted in Sardinia showing that older individuals living in the Sardinian rural areas reported fewer depressive signs than peers residing in urban areas (Carpiniello et al., 1989). Additionally, following previous research (e.g. Jeste et al., 2013), a recent study showed that a facet of resilience known as Optimal Regulation (Alessandri et al., 2007; for an operational definition see “Material” section of this article) significantly concurred in predicting self‐reported depression and psychological well‐being of community‐based older individuals living in the Sardinian Blue Zone (Fastame et al., 2018).

Starting in the 1950s and especially the 1960s to the 1980s, the emigration phenomenon also concerned the Sardinian rural areas (i.e. labour migrants), where according to Rudas et al. (1972) the very poor families decided which members had to leave the island to overcome their economic difficulties (i.e. choosing mainly more ambitious individuals and those showing greater self‐esteem). Even though for decades the Sardinian Regional Government has economically supported the return of Sardinians who emigrate back to their villages of origin, to our knowledge, no studies have been conducted to investigate the effect of return migration (i.e. “return migration is part of the migration process and refers to the act of going back to a place of origin,”; Davies et al., 2011, p. 1) on the mental health of older individuals living in areas of exceptional longevity, like the Sardinian Blue Zone. This is quite surprising, since migration might have represented a positive experience (i.e. improving the economic conditions of the whole family) for older people coming from very poor families. In contrast, the effect of immigration on the mental health of Sardinians has been evaluated across the adult life span in two studies showing controversial evidence. Indeed, it has been pointed out that Sardinians resident on the island self‐reported fewer depressive symptoms than Sardinian adults living in Paris (Carta et al., 2002). In contrast, in a further recent study, Carta et al. (2017) highlighted that Sardinians over the age of 18 who emigrated to Argentina were less likely to be depressed than permanent residents in Sardinia. Additionally, Carta et al. (2002) also showed that Sardinian adults who returned to their Sardinian place of origin after having emigrated for at least 1 year, reported greater dysthymic symptoms than a sample of Sardinian permanent residents (i.e. peers who did not experience migration). However, the authors did not selectively report any specific evidence about the mental health of their older (i.e. over the age of 64) participants with and without a migratory background. This issue is very relevant, because the returnees can face a series of living problems (e.g. disappointed expectations) which, in turn, can negatively impact their mental condition (e.g. increased distress) after they return to the place of origin (Gillespie et al., 2000). Concerning this issue, the only longitudinal study of which we are aware was recently conducted in Germany on the role played by psychological well‐being, like determinants of older migrants' intention to return to their place of origin (Cela & Bettin, 2018). This investigation documented that greater life satisfaction and length of migration diminished return intentions. However, to our knowledge, no studies have been conducted to examine the psychological resources of older migrants after their return home. This is quite surprising, considering that a psychological trait such as resilience is essential to face stressful events like migration experiences (e.g. Klokgieters et al., 2020).

This investigation was mainly aimed at evaluating some dimensions of self‐reported mental health in older individuals living in the Sardinian Blue Zone with and without a return migration experience. Specifically, this study intended to explore whether community‐based older individuals living in an area of exceptional longevity that experienced emigration reported different indexes of mental health than permanent resident peers living in the same geographical spot for their whole life. In addition, the nature of the associations between the years of emigration and perceived mental health measures was also evaluated. Based on the analysis of the literature: (a) compared to the national normative data, participants without a migratory background were expected to report very high psychological well‐being and low depressive symptoms (e.g. Fastame et al., 2015; Fastame, Penna, & Rossetti, 2014) and (b) Sardinians who emigrated were expected to show greater depressive symptoms than peers who had never emigrated (Carta et al., 2002). Due to the lack of relevant evidence about the impact of return migration on resilience and well‐being in adulthood, further a priori hypotheses were not proposed.

## METHODS

### Participants

Forty‐eight community‐based older individuals, 32 males and 16 females (*M*_age_ = 74.5 years, *SD* = 5.2), were recruited in two villages (∼2000 inhabitants in total) of Sardinia's Blue Zone, namely Talana and Urzulei. Specifically, 24 participants having experienced emigration (i.e. emigrated group) in their youth and/or midlife were age, gender, global cognitive functioning, and education‐matched with a subsample of permanent resident participants living in the same geographical area and who spent their whole life in their villages (i.e. permanent resident group). Overall, 29 respondents (16 permanent residents and 13 individuals who experienced emigration) were enrolled at Urzulei, whereas 19 other participants (11 permanent residents and 8 individuals who experienced emigration) were recruited at Talana. All the respondents took voluntarily part in the study and did not receive any economic compensation or other rewards for their participation.

To participate in the study, the following inclusion criteria had to be satisfied: (a) to be community‐based; (b) to be born and resident in the Sardinian Blue Zone; (c) to be a descendant of families living in that geographic spot for a minimum of two previous generations; (d) to be ≥65 years old; (e) to display a score ≥ 20 to the Mini‐Mental State Examination (Folstein et al., 1975) to exclude the occurrence of moderate and severe signs of cognitive deterioration. Additionally, the subsample of Sardinians emigrated encompassed participants that spent at least 1 year in the Italian mainland or abroad and that after this migration experience, returned to live in their place of origin located in the Sardinian Blue Zone.

The place of residence (i.e. Talana vs. Urzulei) was counterbalanced across the participants (χ^2^ = 2.08, *df* = 1, *p* = .15). Moreover, no significant differences were found between the returned emigrated group and the permanent resident one in terms of age (*t*(46) = −.81, *p* = .935), general cognitive functioning (*t*(46) = .21, *p* = .84), and years of formal education (*t*(46) = −.24, *p* = .811). Finally, gender (*χ* ^2^ = 5.333, *df* = 1, *p* = .021) and marital status (*χ* ^2^ = 5.83, *df* = 1, *p* = .016) were not equally distributed across the participants. The unbalanced distribution of old males and females who experienced emigration and took part in the study may be attributed to the fact that in the past, married women of the Sardinian rural and poorest areas remained in their villages looking after their children and parents, while their spouses (usually shepherds) emigrated alone to overcome the economic problems of their families. Table 1 summarises the characteristics of the sample.

**Table 1:** Socio‐demographic characteristics and global cognitive functioning (i.e. MMSE) scores collected from all the participants of the study that spent their whole life in the Sardinian Blue Zone (i.e. permanent resident group) or that emigrated in the Italian mainland or abroad (i.e. emigrated group)

Variable	Permanent resident group	Emigrated group
*n*	24	24
Gender		
Males	16	16
Females	8	8
Age (years)	*M* = 74.5	*M* = 74.6
	(*SD* = 5.4)	(*SD* = 5.2)
Education (years)	*M* = 6.2	*M* = 6.5
	(*SD* = 3.2)	(*SD* = 2.8)
Marital status		
Single/widow	6	7
Married	18	17
MMSE score	*M* = 26.16	*M* = 26.01
	(*SD* = 2.8)	(*SD* = 2.2)
Signs of cognitive decline		
No	20	20
Yes	4	4
Emigration		
In the mainland	0	7
Abroad	0	17
Years of emigration	*M* = 0	*M* = 14.6 years
		(*SD* = 13.9)

### Ethics statement

The study was conducted in accordance with the ethical standards of the institutional research committee and with the 1964 Helsinki Declaration and its later amendments. Written informed consent was given by all participants before participation.

### Materials

Each participant completed the following battery of test and inventories:

The Mini‐Mental State Examination (MMSE; Folstein et al., 1975) was used to objectively evaluate global cognitive functioning. This is a screening test that encompasses 20 items assessing 11 cognitive functions, such as spatio‐temporal orientation, attention, short and long‐term memory. A score ≥ 24 indicated the lack of signs of cognitive impairment (maximum total score = 30). Following Magni et al. (1996), the MMSE score was adjusted for educational attainment and age.

The SODdisfazione dell'Anziano (SODA) Questionnaire (Fastame et al., 2020) was designed to self‐rate the level of personal satisfaction relative to the previous week. The SODA measure includes 14 items, and for each statement, the respondent had to assess his/her level of hedonic well‐being using an 11‐point Likert scale ranging from 0 (*not satisfied*) to 10 (*completely satisfied*). The total score ranges from 0 to 140. Apart from a total life satisfaction index (i.e. SODA‐tot), three further parameters concerning the satisfaction for one's physical and cognitive health (SODA‐health), religious well‐being (i.e. SODA‐rel), and satisfaction for the time spent for leisure activities (i.e. SODA‐time) were calculated. In the current sample, the internal consistency for the overall scale was 0.73.

The Psychological Well‐Being and Ageing Questionnaire (PWBQ; De Beni et al., 2007) was validated to assess different dimensions of psychological well‐being, that is, coping strategies (i.e. PWBQ‐sc), personal satisfaction (i.e. PWBQ‐ps), emotional competence (i.e. PWBQ‐ce) and total well‐being (i.e. PWBQ‐tot) in Italian sexagenarians and older individuals. This self‐report inventory encompasses 37 items, for each statement the participant had to self‐rate the occurrence of the described situation on a Likert scale ranging from 1 (*never*) to 4 (*always*). The total well‐being score ranges from 37 to 148 and according to the Italian normative data, a total score ≥ 115 indicates the greatest level of total psychological well‐being. In the current sample, the internal consistency for the total psychological well‐being score was very good (Cronbach α = .92).

The Center of Epidemiological Studies‐Depression Scale (CES‐D, Radloff, 1977; Italian version, Fava, 1983) was designed to self‐assess the occurrence of distress symptoms. This tool is composed of 20 items, such that for each statement the participant had to rate his/her negative affect during the past week on a 4‐point Likert scale ranging from 0 (*rarely or never*) to 3 (*most days or every day*). The maximum total score is 60. A score ≥ 16 implies the presence of significant depressive symptoms, whereas a score ≥ 23 suggests the occurrence of suspected major depression. In the current sample, Cronbach α was .82.

The Ego Resiliency Scale‐Revised (ER89‐R; Alessandri et al., 2007) was designed to assess two different facets of resilience: Optimal Regulation (i.e. RES‐OR, the set of personal resources enabling the individual to manage negative emotionality) and Openness to Life experiences (i.e. RES‐OL, a facet of ego‐resilience referring to the personal resources used to manage positive emotionality when it is necessary to adapt to adversity). The self‐report inventory is composed of 10 items, for each statement, the respondent had to self‐rate his/her level of agreement on a 7‐point Likert scale ranging from 1 (*does not apply at all*) to 7 (*applies very strongly*). A total score was computed for each subscale. The maximum total score for the RES‐OR subscale is 42, whereas the maximum RES‐OL total score is 28. In the current sample, the alpha for the overall scale was 0.74.

The revised version of the preliminary interview used by Fastame et al. (2021) was designed to collect information about the lifestyle habits and socio‐demographic characteristics of the participants.

### Procedure

Written informed consent had to be provided by each participant before participation in the study. Each respondent was individually tested in a quiet room of his/her own home. After that the MMSE questionnaire was administered, if the occurrence of significant cognitive deterioration were excluded (i.e. a score ≥ 24) or if the clinical signs denoted only the occurrence of very early symptoms associated with suspected cognitive decline (i.e. MMSE score ranging between 23 and 20), the socio‐demographic information of the participant was collected. Then, the presentation order of the psychological inventories assessing different facets of perceived mental health was counterbalanced across the participants according to the Latin Square procedure. To avoid the fatigue effect, each statement was read by the experimenter that also recorded the responses on the response sheets. The assessment of each participant lasted approximately 1 hour.

## RESULTS

First, three separated multivariate analyses of covariance (MANCOVAs) were performed to explore the impact of the experience of emigration (Non‐Migratory group vs. Emigrated group) on psychological well‐being assessed through the PWBQ and SODA inventories and resilience indexes, controlling for the effect of duration of emigration and global general efficiency (i.e. MMSE score).

Relative to the PWBQ condition, the multivariate tests did not show the significant main effects of emigration (Wilks' λ = .868, *df* = 4;41, *p* = .20) and years of emigration (Wilks' λ = .952, *df* = 4;41, *p* = .20), whereas there was the main effect of the covariate MMSE (Wilks' λ = .757, *df* = 4;41, *p* = .020). Overall, no differences were found between the Non‐Migratory and the Emigrated groups in terms of total PWBQ well‐being (*F*(1, 44) = .016, *p* = .342), coping strategies (*F*(1, 44) = .352, *p* = .556), personal satisfaction (*F*(1, 44) = .194, *p* = .662), and emotional competence (*F*(1, 44) = 1.78, *p* = .189). A similar pattern of results was found when the SODA measures were considered. Indeed, the multivariate tests did not show any significant main effect of the experience of emigration (Wilks' λ = .978, *df* = 3;42, *p* = .812), duration of emigration (Wilks' λ = .887, *df* = 3;42, *p* = .166) and global cognitive efficiency (Wilks' λ = .916, *df* = 3;42, *p* = .293). Indeed, no differences were found between the Emigrated and Non‐Migratory groups in terms of total personal satisfaction (*F*(1, 44) = .408, *p* = .527), satisfaction about one's cognitive and physical health (*F*(1, 44) = .054, *p* = .817), satisfaction about the time spent for leisure activities (*F*(1, 44) = .990, *p* = .325), and religious well‐being (*F*(1, 44) = .008, *p* = .931). In contrast, when the resilience indexes were taken into account, the multivariate tests showed the main effect of the experience of emigration (Wilks' λ = .868, *df* = 2;43, *p* = .047), whereas there were not the effects of the covariate MMSE (Wilks' λ = .918, *df* = 3;42, *p* = .159) and duration of emigration (Wilks' λ = .949, *df* = 3;42, *p* = .324). The main effect of the experience of emigration was significant in the RES‐OL (*F*(1, 44) = 6.691, *p* = .013, η^2^ = .132) and total resilience (*F*(1, 44) = 4.856, *p* = .033, η^2^ = .10) conditions but not in the RES‐OR one (*F*(1, 44) = .68, *p* = .414).

Moreover, an analysis of the covariance (ANCOVA) was conducted to examine the effect of the experience of emigration on the self‐reported depression measure, using MMSE and duration of emigration as covariates. The main effects of the experience of emigration (*F*(1, 44) = .135, *p* = .715), global cognitive efficiency (*F*(1, 44) = .817, *p* = .371) and years of emigration (*F*(1, 44) = .383, *p* = .539) were not significant. Table 2 illustrates the mean scores of the Non‐Migratory and Emigrated groups in each mental health condition.

**Table 2:** Psychological Well‐being (i.e. PWBQ), Depression (i.e. CES‐D), Life Satisfaction (i.e. SODA), and resilience mean indexes recorded across the permanent resident and emigrated groups, respectively

Measure	Permanent resident group	Emigrated group
PWBQ		
Total	*M* = 120.38 (*SD* = 13.64)	*M* = 121.79 (*SD* = 15.17)
Personal satisfaction	*M* = 37.50 (*SD* = 4.85)	*M* = 37.88 (*SD* = 4.60)
Coping strategies	*M* = 27.50 (*SD* = 4.10)	*M* = 26.58 (*SD* = 4.97)
Emotional competence	*M* = 32.33 (*SD* = 13.04)	*M* = 33.83 (*SD* = 4.79)
CES‐D	*M* = 14.54 (*SD* = 10.19)	*M* = 12.04 (*SD* = 8.54)
SODA	*M* = 105.08 (*SD* = 16.36)	*M* = 107.16 (*SD* = 16.28)
Total	*M* = 60.33 (*SD* = 9.73)	*M* = 62.42 (*SD* = 9.47)
Health	*M* = 29.92 (*SD* = 9.02)	*M* = 29.3 (*SD* = 9.8)
Time	*M* = 14.83 (*SD* = 6.78)	*M* = 15.42 (*SD* = 6.48)
Religious well‐being resilience		
Total	*M* = 51.67 (*SD* = 7.61)	*M* = 54.71 (*SD* = 5.74)
RES‐OL	*M* = 18.96 (*SD* = 5.11)	*M* = 21.79 (*SD* = 4.46)
RES‐OR	*M* = 32.87 (*SD* = 4.08)	*M* = 33.17 (*SD* = 2.93)

*Note*: Standard deviations are illustrated in parentheses.

Finally, a series of Pearson product–moment correlation coefficients between the duration of emigration expressed in years and perceived depression, psychological well‐being, and resilience were computed. These outcomes are summarised in Table 3.

**Table 3:** Pearson product moment correlation coefficients calculated between length of emigration (i.e. years of emigration), perceived total psychological well‐being (i.e. PWBQ‐tot), personal satisfaction (i.e. PWBQ‐ps), coping strategies (i.e. PWBQ‐sc), emotional competence (i.e. PWBQ‐ce), depression (i.e. CES‐D), total satisfaction (i.e. SODA‐tot), satisfaction about one's cognitive and physical health (i.e. SODA‐health), religious well‐being (i.e. SODA‐rel), satisfaction about time spent for leisure activities (i.e. SODA‐time), and two facets of ego‐resilience, that is, Optimal Regulation (i.e. RES‐OR) and Openness to Life experiences (i.e. RES‐OL), respectively

	Years of emigration	PWBQ‐tot	PWBQ‐ps	PWBQ‐sc	PWBQ‐ce	CES‐D	SODA‐TOT	SODA‐health	SODA‐rel	SODA‐time	RESOR	RESOL
Years of emigration	—											
PWBQ‐tot	−0.032	—										
PWBQ‐ps	−0.025	0.877^***^	—									
PWBQ‐sc	−0.036	0.786^***^	0.505^***^	—								
PWBQ‐ce	−0.012	0.835^***^	0.621^***^	0.592^***^	—							
CES‐D	−0.169	−0.372^**^	−0.466^***^	−0.230	−0.185	—						
SODA‐TOT	−0.043	0.496^***^	0.484^***^	0.423^**^	0.441^**^	−0.370^**^		—				
SODA‐health	0.125	0.372^**^	0.321^*^	0.368^*^	0.321^*^	−0.440^**^	—	0.785^***^	—			
SODA‐rel	0.095	0.019	0.078	0.036	0.016	−0.022	0.369^***^	0.094	—			
SODA‐time	−0.270	0.465^***^	0.456^**^	0.332^*^	0.424^**^	−0.174	0.670^***^	0.270	−0.161	—		
RES‐OR	−0.071	0.525^***^	0.577^***^	0.273	0.331^*^	−0.347^*^	0.270	0.212	−0.127	0.341^*^	—	
RES‐OL	0.047	0.348^*^	0.178	0.315^*^	0.395^**^	−0.108	0.120	0.160	−0.133	0.138	0.314^*^	—

* *p* < .05.

** *p* < .01.

*** *p* < .001.

## DISCUSSION

The main goal of this study was to evaluate the associations between the migration experience and several measures of perceived mental health in a sample of older individuals living in an area of exceptional longevity, such as the Blue Zones. According to Poulain et al. (2004, 2013), these are specific and limited geographical spots characterised by a higher prevalence of centenarians located in Japan (Okinawa), on the Italian island of Sardinia, in Greece (Ikaria), and in Costa Rica (Nicoya Peninsula).

To our knowledge, this is the first investigation aimed at comparing the perceived psychological well‐being and resilience of older permanent residents living in the Sardinian Blue Zone and their peers who had an experience of migration but returned to live in their village of origins (i.e. returnees). Overall, the current outcomes suggest at least three conclusions. First, older participants who emigrated reported better ego resilience than Sardinian permanent residents, and the former group developed greater resources for the management of positive emotionality as a medium to adapt oneself to adverse and stressful events. Thus, extending previous evidence (Gillespie et al., 2000; Klokgieters et al., 2020), it is plausible to hypothesise that resilience is a psychological trait that helps returnees to face the stressful events related both to the migratory experience and the living problems associated with the return to their place of origin. Furthermore, as pointed out in previous studies (Fastame et al., 2018; Jeste et al., 2013), the current outcomes contribute to highlighting that resilience is a protective factor promoting successful ageing.

Additionally, unlike what was reported by Carta et al. (2002) in a sample of Sardinian adults who experienced migration but returned to Sardinia, our returnees did not show greater depressive symptoms compared to a group of permanent old residents who spent their lives in the Sardinian Blue Zone. Instead, in line with previous studies (e.g. Fastame, Penna, & Rossetti, 2014), both our groups (i.e. returnees vs. permanent residents) displayed lower CES‐D scores than the Italian cut‐off (i.e. ≥16) used for the diagnosis of significant depressive symptoms. Overall, the inconsistency between our results and those highlighted by Carta et al. (2002) could be justified by three different reasons. First, our study was focused only on the self‐assessment of negative mood in older individuals, whereas an objective assessment is lacking. In addition, it must be kept in mind that older people in Sardinian rural areas who experienced migrations came from very poor families, therefore, it is plausible to assume that the migration was a positive experience that improved the economic conditions of all family members. Indeed, during the socio‐demographic interview, our returnees stated that they were labour migrants, pointing out that the only reason driving their migration experience was the wish to surmount the economic difficulties of the family and to improve their work status. Furthermore, extending previous studies, it is plausible to hypothesise that older people in the Sardinian Blue Zone belong to a geriatric subpopulation with specific positive psychological characteristics, which, in turn, represent a protective factor for their mental health and their optimal ageing. In particular, this speculation seems to highlight the consistency between the current findings and previous evidence showing that community‐based older people living in the Sardinian Blue zone reported fewer depressive symptoms and greater hedonic and eudaimonic psychological well‐being than peers living in rural areas located in northern Italy and in the Sardinian capital (Fastame et al., 2015, 2021; for a review, see Hitchcott et al., 2018). Concerning this issue, as expected (e.g. Fastame et al., 2015; Fastame, Penna, & Rossetti, 2014), compared to the national cut‐off, our participants, both the returnees and the permanent residents, reported very high levels of general well‐being and personal satisfaction assessed through the PWBQ developed by De Beni et al. (2007) and fewer depressive symptoms evaluated through the CES‐D inventory. Thus, consistently with Fastame, Penna, Rossetti, and Agus (2014), it is plausible to assume that the high level of mental health reported by our participants, even the returnee ones, can be justified by contextual factors. Indeed, our participants were recruited in a socio‐cultural context where collectivistic values prevail and where the older individuals are considered a resource for the community. Additionally, as pointed out by Davies et al. (2011), returning to a supportive socio‐cultural context where the returnees share a set of social, cultural, and linguistic norms with permanent residents can favour the improvement of mental health in those who experienced migration. Extending this, a recent study conducted among older individuals of the Sardinian Blue Zone and peers living in the Sardinian capital (i.e. Cagliari) documented that the former group reported more flourishing (i.e. a facet of eudaimonic well‐being, implicating having a purposeful and meaningful life, being engaged with an active life, and one's satisfaction with social relationships) and greater satisfactions with their relationships with family members and friends than the group of the participants enrolled in the urban area (Fastame et al., 2021).

However, some limitations of this study need to be discussed. To our knowledge, this is the first study conducted to evaluate the associations between the migration experience and the perceived mental health of older individuals ageing well who decided to return to their place of origin. Therefore, caution is needed in generalising the current findings to older individuals living in other Blue Zones, in other Sardinian areas or elsewhere. A further limitation is that this study had a very small sample size, was cross‐sectional and correlational in design, and relied on mono‐informants who completed a limited number of self‐report questionnaires. Therefore, future research is necessary to replicate this study in other socio‐cultural contexts (e.g. other Blue Zones), using larger samples to collect data from multiple informants (e.g. returnees, spouses, children, general practitioners), including participants who experienced migration for different reasons (e.g. wars, famine), and adopting a longitudinal approach.
